# Supplementary figures and images for: A microRNA generated via lysosomal processing of ribosomal RNA suppresses proinflammatory responses
Source: Life Sci Alliance. 2026 May 4;9(7):e202503536. doi: 10.26508/lsa.202503536 (PMC13139743; doi:10.26508/lsa.202503536)

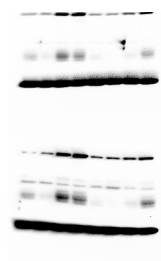

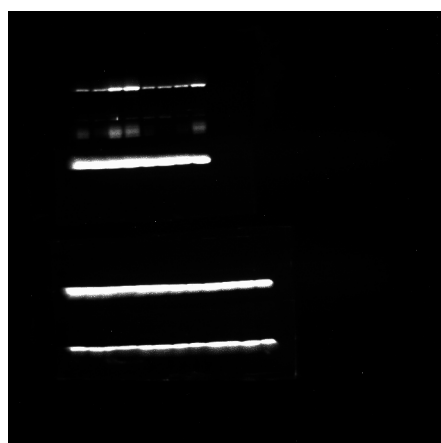

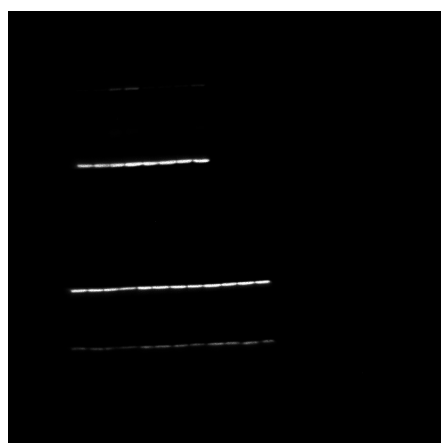

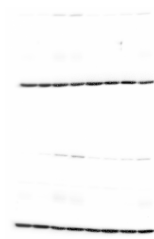

Supplement: Supplementary file 5 [file LSA-2025-03536_SdataF7.pdf]
