# Supplementary material for: A microRNA generated via lysosomal processing of ribosomal RNA suppresses proinflammatory responses
Source: Life Sci Alliance. 2026 May 4;9(7):e202503536. doi: 10.26508/lsa.202503536 (PMC13139743; doi:10.26508/lsa.202503536)
Supplement: Supplemental Data 2. — Fold change and FDR values relevant for Fig 8A. [file LSA-2025-03536_Supplemental_Data_2.docx]

**Supplementary Data 2**

**Fold change and FDR values relevant for Fig 8A**.

The numbers below represent average log_2_ fold change values obtained from 3 biological repeats. The script on the bottom refers to reference samples.

The numbers below represent false discovery rates (FDR), determined by statistical analysis of variance (ANOVA). The script on the bottom refers to reference samples.
